# Supplementary material for: Effect of rest, post-rest transport duration, and conditioning on performance, behavioural, and physiological welfare indicators of beef calves
Source: PLoS One. 2022 Dec 1;17(12):e0278768. doi: 10.1371/journal.pone.0278768 (PMC9714911; doi:10.1371/journal.pone.0278768)
Supplement: S1 File — (A) Meal duration, (B) meal frequency, (C) meal size, (D) feeding intake, (E) feeding rate, and (F) feeding time. (DOCX) [file pone.0278768.s001.docx]

S1. File

Feeding behaviour

Meal size

A conditioning × transport × rest (nested in time) effect (*p* < 0.01) was observed for meal size. The C-R0-T4 calves were characterized by greater (*p* ≤ 0.03) mean meal size than N-R0-T4 calves on d 1, 2, 4, and 10. The C-R0-T15 calves were characterized by greater (*p* ≤ 0.03) mean meal size than N-R0-T15 calves on d 1 and 4. The C-R8-T15 calves were characterized by greater (*p* ≤ 0.03) mean meal size than N-R8-T15 calves on d 1 and 2. At d 2, N-R8-T15 calves were characterized by greater (*p* < 0.01) mean meal size than N-R8-T4 calves. At d 3, C-R8-T4 calves were characterized by greater (*p* = 0.04) meal size than N-R0-T4 calves. At d 4, C-R8-T4 calves were characterized by greater (*p* ≤ 0.02) mean meal size than N-R8-T4 calves. At d 12, C-R0-T4 and N-R8-T4 calves were characterized by greater (*p* < 0.01) mean meal size than C-R8-T4 and C-R8-T4 calves, respectively. At d 13, C-R0-T4 calves were characterized by greater (*p* = 0.02) mean meal size than C-R8-T4 calves. At d 25, C-R8-T15 calves were characterized by greater (*p* < 0.01) mean meal size than C-R8-T4 calves. At d 26, N-R0-T4 and N-R8-T4 calves had greater (*p* < 0.01) meal size than C-R0-T4 and C-R8-T4 calves, respectively. Higuchi H., Katoh N., Miyamoto T., Uchida E., Yuasa A. and Takahashi K., 1994. Dexamethasone-induced haptoglobin release by calf liver parenchymal cells. *American Journal of Veterinary Research*, **55**, 1080–1085 Higuchi H., Katoh N., Miyamoto T., Uchida E., Yuasa A. and Takahashi K., 1994. Dexamethasone-induced haptoglobin release by calf liver parenchymal cells. *American Journal of Veterinary Research*, **55**, 1080–1085 Higuchi H., Katoh N., Miyamoto T., Uchida E., Yuasa A. and Takahashi K., 1994. Dexamethasone-induced haptoglobin release by calf liver parenchymal cells. *American Journal of Veterinary Research*, **55**, 1080–1085

Meal duration

A conditioning × transport × rest (nested in time) effect (*p* < 0.01) was observed for meal duration. At d 9, N-R0-T15 calves were characterized by greater (*p* < 0.01) mean meal duration than N-R0-T4 calves. On d 12, N-R8-T4 calves were characterized by greater (*p* ≤ 0.03) mean meal duration than N-R0-T4, C-R8-T4, and N-R8-T15 calves. At d 13, N-R8-T4 calves were characterized by greater (*p* = 0.03) mean meal duration than C-R8-T4 calves. At d 14, N-R8-T4 calves were characterized by greater (*p* ≤ 0.01) mean meal duration than C-R8-T4 and N-R0-T4 calves. At d 21, C-R0-T15 calves were characterized by greater (*p* < 0.01) mean meal duration than N-R0-T15 calves. At d 25, C-R8-T15 calves were characterized by greater (*p* = 0.01) mean meal duration than C-R8-T4 calves. At d 26, N-R0-T4 calves were characterized by greater (*p* = 0.01) mean meal duration than C-R0-T4 calves.

Meal frequency

A conditioning × transport × rest (nested in time) effect (*p* < 0.01) was observed for meal frequency. At d1, C-R0-T4 and C-R0-T15 calves were characterized by greater (*p* < 0.01) mean meal frequency than N-R0-T4 and N-R0-T15 calves, respectively, while C-R8-T4 calves were characterized by greater (*p* < 0.01) mean meal frequency than N-R8-T4 and C-R8-T15 calves. At d2, C-R8-T15 calves were characterized by greater (*p* = 0.02) meal frequency than N-R8-T15 calves. At d 12, C-R8-T4 calves were characterized by greater (*p* < 0.01) mean meal frequency than C-R0-T4 and N-R8-T4 calves, while N-R0-T4 and N-R8-T15 calves were characterized by greater (*p* = 0.04) mean meal frequency than N-R8-T4 calves. At d 25, C-R8-T4 calves were characterized by greater (*p* < 0.01) mean meal frequency than C-R8-T15 calves. At d 26, C-R0-T4 calves were characterized by greater (*p* < 0.01) mean meal frequency than N-R0-T4 calves.

Feeding rate

A conditioning × transport × rest (nested in time) effect (*p* < 0.01) was observed for feeding rate. The C-R0-T4 calves were characterized by greater (*p* < 0.01) mean feeding rate than N-R0-T4 on d 1, 2, 3, 4 and 5. The C-R0-T15 calves were characterized by greater (*p* < 0.01) mean feeding rate than N-R0-T15 on d 1, 2, and 4. The C-R8-T15 calves were characterized by greater (*p* < 0.01) mean feeding rate than N-R8-T15 on d 1, 2, and 3. At d 1, N-R8-T15 calves were characterized by greater (*p* < 0.01) mean feeding rate than N-R8-T4 calves, while C-R8-T4 calves were characterized by greater (*p* ≤ 0.03) mean feeding rate than N-R8-T4 and C-R8-T15 calves. At d 2, C-R8-T4 calves were characterized by greater (*p* < 0.01) mean feeding rate than N-R8-T4 calves, respectively. At d 3, C-R8-T4 calves were characterized by greater (*p* < 0.01) feeding rate than N-R8-T4. At d 4, N-R0-T15 and C-R8-T4 calves were characterized by greater (*p* < 0.05) mean feeding rate than N-R8-T15 and N-R8-T4 calves.

Feeding time

A conditioning × transport × rest (nested in time) effect (*p* < 0.01) was observed for feeding time. At d 1, C-R0-T4 calves were characterized by greater (*p* < 0.01) mean feeding time than N-R0-T4 calves, while C-R8-T4 calves were characterized by greater (*p* < 0.01) mean feeding time than N-R8-T4 and C-R8-T15 calves. At d 2, C-R8-T15 calves were characterized by greater (*p* < 0.01) feeding time than N-R8-T15 calves. At d 4, C-R0-T15 and N-R8-T15 calves were characterized by greater (*p* < 0.01) mean feeding time than N-R0-T15. At d 9, C-R0-T4, N-R0-T15, and N-R8-T4 calves were characterized by greater (*p* < 0.01) mean feeding time than N-R0-T4 calves. At d 14, N-R8-T15 calves were characterized by greater (*p* = 0.05) feeding time than C-R8-T15 calves. At d 23, C-R8-T4 calves were characterized by greater (*p* = 0.05) feeding time than C-R0-T4 calves.

Feeding intake

A conditioning × transport × rest (nested in time) effect (*p* < 0.01) was observed for feeding intake. The C-R0-T4 calves were characterized by greater (*p* < 0.01) mean feeding intake than N-R0-T4 on d 1, 2, 3, 4, and 9. The C-R0-T15 calves were characterized by greater (*p* < 0.01) mean feeding intake than N-R0-T15 at d 1, 2, 4, and 5. The C-R8-T4 calves were characterized by greater (*p* < 0.01) mean feeding intake than N-R8-T4 calves on d 1, 2, 3, and 4. The C-R8-T15 calves were characterized by greater (*p* ≤ 0.03) mean feeding intake than N-R8-T15 calves at d 1, 2, and 3. At d1, the C-R8-T4 and N-R8-T15 calves were characterized by greater (*p* ≤ 0.03) mean feeding intake than C-R8-T15 and N-R8-T4 calves. At d 4, N-R8-T15 calves were characterized by greater (*p* = 0.05) mean feeding intake than N-R0-T15 calves. At d 9, C-R0-T4 calves were characterized by greater (*p* < 0.01) mean feeding intake than C-R8-T4 calves.

Higuchi H., Katoh N., Miyamoto T., Uchida E., Yuasa A. and Takahashi K., 1994. Dexamethasone-induced haptoglobin release by calf liver parenchymal cells. *American Journal of Veterinary Research*, **55**, 1080–1085S1 Fig 1. Least squares-means of feeding behavior of conditioned (C) and non-conditioned (N), calves rested for 0 (R0) or 8 (R8) h and transported for an additional 4 (T4) or 15 (T15) h.

1. Meal duration, (B) meal frequency, (C) meal size, (D) feeding intake, (E) feeding rate, and (F) feeding time.
