# Supplementary material for: Effect of rest, post-rest transport duration, and conditioning on performance, behavioural, and physiological welfare indicators of beef calves
Source: PLoS One. 2022 Dec 1;17(12):e0278768. doi: 10.1371/journal.pone.0278768 (PMC9714911; doi:10.1371/journal.pone.0278768)
Supplement: S1 Table — (DOCX) [file pone.0278768.s002.docx]

S1 Table. Generalized linear mixed modelling (SAS POC GLIMMIX statements) indicating the response variable, the selected distribution, the link function, and the selected structure of the covariance matrix.

| **Experimental variable** | **Distribution** | **Link function** | **Covariance structure** |
| --- | --- | --- | --- |
| NEFA | GAMMA | Log | AR(1) |
| HP | T | Identity | AR(1) |
| SAA | LOGN | Identity | AR(1) |
| Serum cortisol | GAMMA | Log | VC |
| L-Lactate | N | Identity | VC |
| CK | GAMMA | Log | AR(1) |
| Weight | LOGN | Identity | ARH(1) |
| ADG w 1 | T | Identity | - |
| ADG w 2 | LOGN | Identity | - |
| ADG d 14 - 28 | GAMMA | Log | - |
| Shrink 1 | LOGN | Identity | - |
| Shrink 2 | LOGN | Identity | - |
| Feeding Time | LOGN | Identity | VC |
| Feeding Intake | LOGN | Identity | VC |
| Feeding Rate | LOGN | Identity | VC |
| Meal frequency | LOGN | Identity | VC |
| Meal duration | LOGN | Identity | VC |
| Meal size | GAMMA | Log | VC |
| Flight Speed | GAMMA | Log | AR(1) |
| DMI | GAMMA | Log | - |
| Standing bout duration d1-d5 | GAMMA | Log | - |
| Lying bout duration d1-d5 | GAMMA | Log | - |
| Standing percentage d1-d5 | GAMMA | Log | - |
| Lying percentage d1-d5 | GAMMA | Log | - |
| Attitude score | T | Identity | CSH |
| Lameness score | T | Identity | VC |
| WBC | LOGN | Identity | VC |
| Granulocytes | LOGN | Identity | AR(1) |
| HCT | LOGN | Identity | VC |
| Standing % 20h | GAMMA | Log | - |
| Standing % 4h | GAMMA | Log | - |
| Standing % 8h rest | GAMMA | Log | - |
