# Supplementary material for: Effect of rest, post-rest transport duration, and conditioning on performance, behavioural, and physiological welfare indicators of beef calves
Source: PLoS One. 2022 Dec 1;17(12):e0278768. doi: 10.1371/journal.pone.0278768 (PMC9714911; doi:10.1371/journal.pone.0278768)
Supplement: S2 Table — (DOCX) [file pone.0278768.s003.docx]

S2 Table. Least squares-means (± upper and lower limits at 95% confidence) observed values of production and behavioural parameters of conditioned (C) and non-conditioned (N) calves rested for 0 (R0) or 8 (R8) h and transported for an additional 4 (T4) and (T15) h^1^

|  | Treatment^2^ | | | | | | | |  |  | *p*-value^3^ | | | | |
| --- | --- | --- | --- | --- | --- | --- | --- | --- | --- | --- | --- | --- | --- | --- | --- |
| *Item* | C-R0-T4 | C-R8-T4 | N-R0-T4 | N-R8-T4 | C-R0-T15 | C-R8-T15 | N-R0-T15 | N-R8-T15 | Lower | Upper | Con | Tr | Con×Res×Tr | Time(Res) | C×Tr×Time(Res) |
| BW, kg | 240 | 238 | 228 | 229 | 231 | 240 | 221 | 226 | 221 | 2 | <0.01 | 0.15 | 0.68 | <0.01 | 0.62 |
| Shrink 20h, % | 9.4 | 10.1 | 4.5 | 3.9 | 9.7 | 10.2 | 3.8 | 4.0 | 5.78 | 8.33 | <0.01 | 0.68 | 0.39 | - | 0.30 |
| Shrink 4h, % | 1.9 | 3.2 | 1.4 | 1.8 | - | - | - | - | 1.61 | 2.69 | <0.01 | - | - | - | 0.14 |
| Shrink 15h, % | - | - | - | - | 4.0 | 6.9 | 3.3 | 4.2 | 3.9 | 5.5 | <0.01 | - | - | - | <0.01 |
| ADG w1, kg/day | -1.39 | -1.15 | 1.43 | 1.81 | -1.40 | -1.93 | 1.37 | 1.33 | -0.61 | 0.63 | <0.01 | 0.61 | 0.09 | - | 0.49 |
| ADG w2,kg/day | 0.59 | 0.50 | 0.48 | 0.46 | 0.46 | 0.56 | 0.52 | 0.76 | 0.31 | 0.78 | 0.66 | 0.26 | 0.19 | - | 0.79 |
| ADG d14-28, kg/day | 1.05 | 0.78 | 0.89 | 0.71 | 1.10 | 0.72 | 0.69 | 0.67 | 0.64 | 1.07 | <0.01 | 0.17 | 0.18 | - | 0.20 |
| DMI, kg/d/head | 5.33 | 5.50 | 3.61 | 3.75 | 4.70 | 5.29 | 3.34 | 3.20 | 3.84 | 4.88 | <0.01 | 0.01 | 0.74 | <0.01 | 0.98 |
| Meal size, kg/meal | 0.98 | 0.87 | 0.81 | 0.81 | 0.98 | 0.95 | 0.79 | 0.81 | 0.79 | 0.97 | <0.01 | 0.30 | 0.61 | <0.01 | <0.01 |
| Meal duration, min/meal | 14.2 | 13.5 | 13.4 | 15.9 | 15.1 | 14.8 | 14.3 | 14.7 | 13.5 | 15.6 | 0.48 | 0.02 | 0.02 | <0.01 | <0.01 |
| Meal frequency meals/day | 14.4 | 14.5 | 13.2 | 12.8 | 14.2 | 12.6 | 13.7 | 14.1 | 12.4 | 15.1 | 0.01 | 0.62 | 0.15 | <0.01 | <0.01 |
| Feed intake, kg/day | 7.6 | 6.7 | 5.7 | 5.5 | 7.5 | 6.4 | 5.6 | 6.1 | 5.5 | 7.5 | <0.01 | 0.53 | 0.47 | <0.01 | <0.01 |
| Feeding rate, g/min | 228 | 198 | 169 | 162 | 224 | 189 | 168 | 182 | 162.6 | 222.3 | <0.01 | 0.53 | 0.47 | <0.01 | <0.01 |
| Feeding time, min/day | 205 | 196 | 177 | 203 | 214 | 186 | 196 | 207 | 176.1 | 222.5 | 0.07 | 0.08 | 0.14 | <0.01 | <0.01 |
| Standing 20h, % | 93.6 | 94.3 | 89.7 | 92.6 | 92.9 | 94.3 | 93.9 | 88.9 | 87.5 | 97.8 | 0.04 | 0.87 | 0.44 | <0.01 | 0.01 |
| Standing 8h rest, % | - | 36.0 | - | 40.0 | - | 42.8 | - | 53.2 | 36.4 | 50.7 | 0.38 | 0.21 | 0.49 | - | 0.02 |
| Standing 4h, % | 95.0 | 62.4 | 87.0 | 47.5 | - | - | - | - | 47.1 | 113.1 | 0.47 | - | 0.68 | <0.01 | - |
| Standing 15h, % | - | - | - | - | 77.8 | 95.4 | 77.9 | 93.6 | 78.8 | 94.3 | 0.77 | - | 0.83 | <0.01 | - |
| Standing d1-5, % | 34.4 | 32.6 | 33.1 | 31.4 | 33.0 | 29.4 | 32.6 | 28.5 | 29.4 | 34.6 | 0.45 | 0.02 | 0.24 | <0.01 | 0.32 |
| Lameness score | -0.01 | 0.00 | -0.01 | 0.02 | 0.02 | 0.00 | 0.00 | 0.02 | -0.04 | 0.05 | 0.69 | 0.37 | 0.74 | 0.18 | 0.41 |
| Attitude score | 0.00 | 0.03 | 0.06 | 0.07 | 0.00 | 0.02 | 0.01 | 0.03 | -0.02 | 0.08 | 0.08 | 0.15 | 0.69 | 0.14 | 0.94 |

Scheffe *p*-values are presented in the table.

^1^Values in the table represent the means of body weight (BW), ADG and flight speed for LO1, UN1, LO2, UN2, 1, 2, 3, 5, 14 and 28 d; the means of shrink 1 for UN1 and shrink 2 for UN2, the means for feed refusals for d 0, 1, and 2, and week 1, 2, and 3, the means of meal size, meal duration, meal frequency, feed intake, feeding rate, feeding time for d 0 to d 28; the means for UN1 and UN2 for attitude score.

^2^ Conditioning: C: conditioned and N: non-conditioned calves. Rest stop: R0: no rest and R8: 8 h of rest. Post-rest transport: 4 (T4) and 15 (T15) h of transport.

^3^ Con: conditioning. Res: rest. Tr: transport
