# Supplementary material for: Effect of rest, post-rest transport duration, and conditioning on performance, behavioural, and physiological welfare indicators of beef calves
Source: PLoS One. 2022 Dec 1;17(12):e0278768. doi: 10.1371/journal.pone.0278768 (PMC9714911; doi:10.1371/journal.pone.0278768)
Supplement: S3 Table — (DOCX) [file pone.0278768.s004.docx]

S3 Table. Least squares-means (± upper and lower limits at 95% confidence) of physiologic parameters of conditioned (C) and non-conditioned (N) calves rested for 0 (R0) or 8 (R8) h and transported for an additional 4 (T4) and (T15) h^1^

|  | Treatment^2^ | | | | | | | |  |  | *p* –value^3^ | | | | |
| --- | --- | --- | --- | --- | --- | --- | --- | --- | --- | --- | --- | --- | --- | --- | --- |
| *Item* | C-R0-T4 | C-R8-T4 | N-R0-T4 | N-R8-T4 | C-R0-T15 | C-R8-T15 | N-R0-T15 | N-R8-T15 | Lower | Upper | Con | Tr | Cond×Res×Tr | Time(Re) | C×Tr×Time(Res) |
| Cortisol, ng/mL | 28.5 | 31.0 | 16.9 | 18.1 | 31.1 | 27.3 | 20.4 | 17.8 | 20.4 | 27.9 | <0.01 | 0.56 | 0.20 | <0.01 | 0.99 |
| SAA, ng/mL | 77 | 157 | 384 | 382 | 163 | 158 | 449 | 310 | 160 | 423 | <0.01 | 0.25 | 0.11 | <0.01 | 0.37 |
| HP, mg/mL | 0.08 | 0.19 | 0.33 | 0.25 | 0.13 | 0.26 | 0.31 | 0.32 | 0.02 | 0.44 | <0.01 | 0.33 | 0.82 | <0.01 | 0.31 |
| L-lactate, mM | 1.3 | 1.2 | 0.7 | 0.9 | 1.3 | 1.1 | 0.9 | 0.8 | 0.85 | 1.20 | <0.01 | 0.64 | 0.43 | <0.01 | 0.70 |
| NEFA, mmol/L | 0.20 | 0.21 | 0.29 | 0.25 | 0.28 | 0.19 | 0.37 | 0.29 | 0.18 | 0.37 | <0.01 | 0.05 | 0.78 | <0.01 | 0.35 |
| CK, U/L | 10.6 | 12.0 | 20.7 | 21.2 | 11.7 | 11.6 | 24.3 | 23.9 | 7.98 | 36.27 | <0.01 | 0.88 | 0.78 | <0.01 | 0.02 |
| HCT, % | 30.3 | 31.5 | 30.7 | 31.2 | 31.6 | 29.4 | 32.3 | 31.6 | 29.5 | 32.8 | 0.16 | 0.60 | 0.11 | <0.01 | 0.56 |
| WBC, ×10^3^/µL | 10.2 | 9.7 | 10.6 | 9.5 | 9.9 | 10.7 | 11.0 | 10.5 | 9.1 | 11.5 | 0.59 | 0.08 | 0.44 | <0.01 | 0.56 |
| Granulocytes, 10^3^/µL | 2.4 | 2.3 | 3.2 | 2.8 | 2.4 | 2.7 | 3.1 | 2.9 | 2.02 | 3.7 | 0.01 | 0.29 | 0.87 | <0.01 | 0.89 |

Scheffe *P*-values are presented in the table.

^1^Values in the table represent the mean of LO1, UN1, LO2, UN2, d 1, 2, 3, 5, 14 and 28 of cortisol, serum amyloid A (SAA), haptoglobin (HP), L-lactate, non-estrified fatty acids (NEFA), creatine kinase, osmolality, hematocrit (HCT), white blood cells (WBC), and granulocytes.

^2^ Conditioning: C: conditioned and N: non-conditioned calves. Source: R: ranch direct and A: auction market calves. Rest stop: 0 h: no rest and 8 h: 8 h of rest.

^3^ Con: conditioning. Res: rest. Tr: transport.
